# Balancing Ethics and Innovation: Can Artificial Intelligence Safely Transform Emergency Surgery? A Narrative Perspective

**DOI:** 10.3390/jcm14093111

**Published:** 2025-04-30

**Authors:** Belinda De Simone, Genevieve Deeken, Fausto Catena

**Affiliations:** 1 Department of Emergency and Digestive Minimally Invasive Surgery, Infermi Hospital, AUSL Romagna, 47921 Rimini, Italy; 2 Department of Theoretical and Applied Sciences, Campus University, Novedrate, 22060 Como, Italy; 3 Department of Global Public Health Global Studies, University of Virginia, Charlottesville, VA 22903, USA; genevievedeeken5@gmail.com; 4 Department of Emergency and General Surgery, Level I Trauma Center, Bufalini Hospital, 474521 Cesena, Italy; faustocatena@gmail.com; 5 Alma Mater Studiorum, University of Bologna, 40100 Bologna, Italy

**Keywords:** artificial intelligence, machine learning, ethics, surgery, emergency surgery, healthcare, transparency, accountability, black box effect

## Abstract

**Background:** Artificial intelligence (AI) is increasingly shaping the landscape of emergency surgery by offering real-time decision support, enhancing diagnostic accuracy, and optimizing workflows. However, its implementation raises significant ethical concerns, particularly regarding accountability, transparency, patient autonomy, and bias. **Objective:** This perspective paper, grounded in a narrative review, explores the ethical dilemmas associated with AI in emergency surgery and proposes future directions for its responsible and equitable integration. **Methods:** A comprehensive narrative review was conducted using PubMed, Scopus, Web of Science, and Google Scholar, covering the literature published from January 2010 to December 2024. We focused on peer-reviewed articles discussing AI in surgical or emergency care and highlighting ethical, legal, or regulatory issues. A thematic analysis was used to synthesize the main ethical challenges. **Results:** Key ethical concerns identified include issues of accountability in AI-assisted decision-making, the “black box” effect and bias in algorithmic design, data privacy and protection, and the lack of global regulatory coherence. Thematic domains were developed around autonomy, beneficence, justice, transparency, and informed consent. **Conclusions:** Responsible AI implementation in emergency surgery requires transparent and explainable models, diverse and representative datasets, robust consent frameworks, and clear guidelines for liability and oversight. Interdisciplinary collaboration is essential to align technological innovation with patient-centered and ethically sound clinical practice.

## 1. Introduction

The rapid advancement of artificial intelligence (AI)-driven technologies is transforming the field of surgery, with emergency surgery emerging as a key area where AI can provide significant clinical benefits. In clinical practice, the Predictive OpTimal Trees in Emergency Surgery Risk (POTTER) calculator, an AI-based tool, has demonstrated superior accuracy in predicting postoperative mortality and complications compared to surgeons’ assessments [1]. Additionally, the development of AI-powered applications like POTTER-Intensive Care Unit (ICU), which predicts the need for ICU admission after emergency surgery, exemplifies how AI can assist in triaging patients and potentially reduce failure-to-rescue rates [2]. AI-based systems are being leveraged to process vast amounts of patient data, assist in decision-making, predict complications, and optimize surgical workflows [3]. However, the unpredictable nature of emergency settings introduces ethical challenges that require careful consideration.

**Figure 1:**
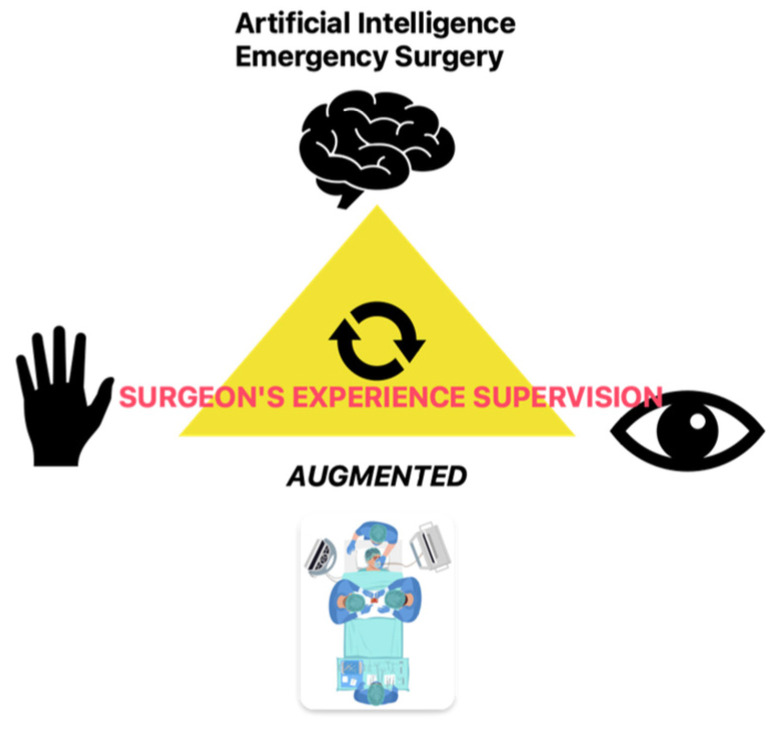
Artificial intelligence emergency surgery: it is the result of the augmented eye, brain, and hand provided by AI tools, under the surgeon’s experience supervision.

The balance between the autonomy of AI-driven tools and human responsibility in making decisions or performing a surgical procedure may seem unclear. A core issue in AI-assisted emergency surgery is maintaining human oversight while leveraging AI’s capabilities, as shown in Figure 1 [4]. In high-pressure, time-sensitive environments where rapid decision-making is required, AI must function as a supportive tool rather than a replacement for human clinical judgment [4,5]. The question remaining is this: “Can AI improve outcomes without undermining ethical standards and human responsibility in surgical decision-making?”

Despite the growing body of literature on AI in surgery, relatively few studies have focused on the specific ethical, legal, and regulatory concerns associated with AI integration in emergency settings. Issues such as informed consent under urgent conditions, data privacy in perioperative environments, algorithmic bias, and lack of explainability pose complex dilemmas. Moreover, the acceleration of technological development has outpaced the establishment of robust frameworks for ethical governance, transparency, and accountability.

This paper aims to explore these challenges by providing a narrative review of the key ethical and regulatory issues surrounding the implementation of AI in emergency surgery and to provide recommendations for clinical practice. Rather than focusing on a quantitative synthesis, we adopt a thematic and critical lens to identify recurring concerns, unresolved questions, and areas requiring further research, and discuss the critical issues related to the emergency setting.

## 2. Methods

This paper is based on a narrative review of the literature, with the aim of identifying and analyzing the most relevant ethical concerns and governance challenges related to AI integration in emergency surgery.

We conducted a search of the literature across the PubMed, Scopus, Web of Science, and Google Scholar databases. The search strategy included the following terms, alone or in combination: “artificial intelligence”, “machine learning”, “computer vision”, “digital surgery”, “emergency surgery”, “healthcare”, “ethics”, “bioethics”, “informed consent”, “black box effect”, “data privacy”, and “regulatory frameworks”.

In our analysis, we included peer-reviewed articles published in English between January 2010 and December 2024, focused on AI technologies in surgical or emergency settings, addressing at least one ethical, legal, or governance issue related to AI use.

Commentaries, non-peer-reviewed materials, editorials, and articles lacking focus on ethical or regulatory aspects were excluded.

An initial pool of 387 articles was identified. After removing duplicates and applying inclusion/exclusion criteria, 112 full-text articles were assessed, and 67 were selected for final inclusion.

**Figure 2:**
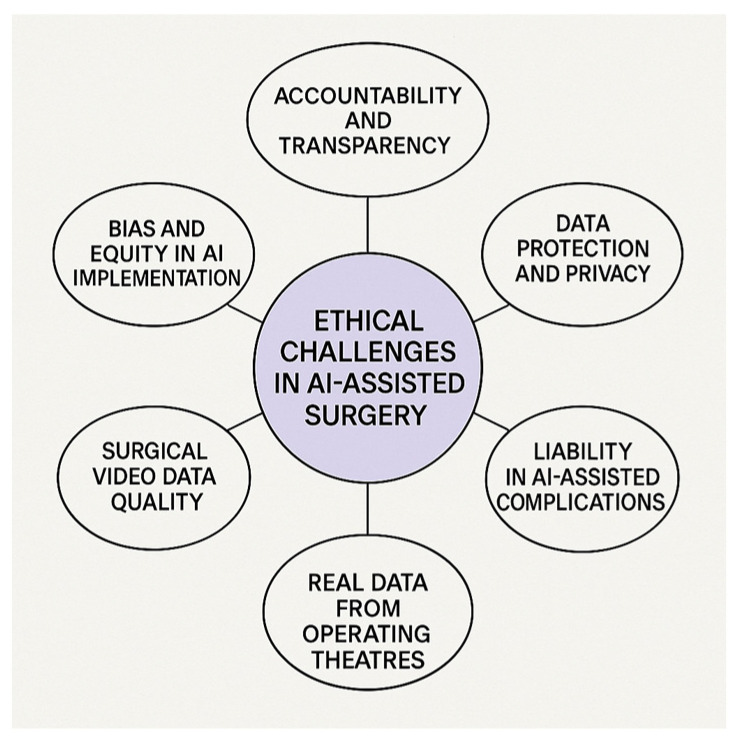
Key ethical challenges in AI-assisted emergency surgery. The figure presents six main ethical themes identified through thematic analysis, providing a conceptual framework for the discussion in this review.

We employed thematic analysis based on Braun and Clarke’s six-phase approach: (1) familiarization with the data; (2) generation of initial codes; (3) searching for themes; (4) reviewing themes; (5) defining and naming themes; and (6) writing up the findings. Key ethical concerns were categorized into six main themes, which structure the core of the discussion presented in this manuscript, as shown in Figure 2 [6].

This narrative review did not involve quantitative synthesis or formal quality appraisal of studies, in line with its exploratory and integrative nature.

## 3. Results

A total of 67 sources were deemed eligible for review. The thematic analysis led to the identification of six major ethical domains: accountability, transparency, data quality, autonomy, liability, and privacy and informed consent. These themes emerged consistently across articles addressing AI integration in surgical and emergency settings, and were selected based on frequency of appearance, depth of ethical discussion, and relevance to acute care contexts.

Notably, while most articles highlighted the promise of AI in enhancing clinical decision-making, few offered detailed solutions for managing associated risks. Many sources emphasized the importance of explainability and fairness, but lacked a unified framework or consensus on implementation. Discrepancies were also observed between regions, with European and North American authors focusing more on legal governance, while others prioritized technical development. These inconsistencies reflect the urgent need for harmonized ethical standards and were critically considered in the formulation of our discussion and recommendations.

To provide a clearer overview of the literature analyzed, Table 1 summarizes the key studies included in this narrative review, their characteristics, and the primary ethical themes discussed.

## 4. Ethical Considerations in AI-Assisted Emergency Surgery: Summary of Evidence and Discussion of Critical Issues for Implementation

Ethical concerns in AI-assisted surgery primarily revolve around informed consent, privacy protection, trust, and potential legal implications [13].

**Figure 3:**
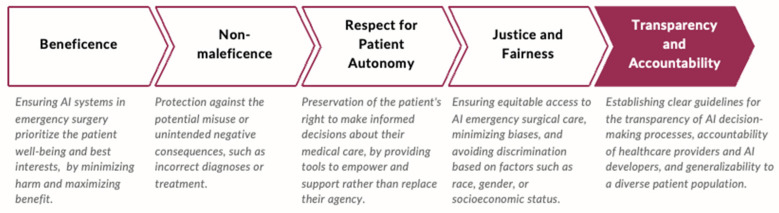
Ethical principles guiding AI integration in surgical care. This framework illustrates how the core bioethical principles—beneficence, non-maleficence, autonomy, and justice—inform patient-centered decision-making when adopting AI technologies in surgery.

The ethical framework guiding the adoption of new surgical technologies is typically structured around four core principles: beneficence, non-maleficence, autonomy, and justice, as summarized in the Figure 3 [14].

However, the ethical landscape of AI in surgery—particularly regarding autonomous and semi-autonomous actions—remains uncertain. This is largely due to the fact that AI-driven procedures could be executed through surgical robots already approved for human use, rather than AI operating independently inside the patient’s body. While robotic arms will deliver AI-powered movements, the AI itself will function externally. Regulatory approval for technologies that do not directly interact with a patient’s internal anatomy is often more straightforward compared to those that do [15].

A current example of an autonomous surgical device already implemented in surgical practices is the use of AI-driven automatic staplers to perform intestinal anastomosis; this device is capable of adjusting stapling speed according to the thickness of tissues using by built-in sensors [16].

As AI-driven automation advances, the question arises of whether more rigorous regulatory processes will be required for increasingly autonomous surgical systems, even if the AI system remains external to the patient.

From an ethical standpoint, the experiences of surgeons and patient expectations in AI-assisted surgery can be categorized into five key areas: rescue, proximity, ordeal, aftermath, and presence [4]. Additionally, experts identified six fundamental ethical concerns in this field: reliability of robotic and AI systems; respect for patient privacy and data protection; use of comprehensive and unbiased datasets; transparency and recognition of AI limitations; equity in healthcare access—avoiding the exacerbation of disparities; and AI as a tool for enhancing surgical education and training [4].

As AI technologies continue to evolve in surgery, addressing these ethical challenges will be crucial in ensuring safe, equitable, and transparent implementation.

### 4.1. Accountability and Transparency

One of the primary ethical concerns surrounding AI in surgery is accountability. If an AI-driven system contributes to a medical error or adverse outcome, determining responsibility becomes complex [7,17]. Traditional medical malpractice frameworks rely on the concept of human agency, which AI challenges by introducing automated decision-making elements. The legal and ethical framework must establish clear accountability mechanisms that delineate human versus machine responsibility in clinical decision-making [18].

The potential for AI-driven decisions to contribute to adverse outcomes depends on the quality, completeness, and representativeness of the datasets used to train AI models. Many current algorithms are built on retrospective or non-standardized data, often lacking input from diverse patient populations or reflecting the unique dynamics of emergency care environments. This “data quality gap” can result in biased or non-generalizable predictions, especially when applied to underrepresented clinical scenarios [7,17,18].

Transparency and interpretability of AI-driven decision-making are essential to fostering trust among healthcare providers and patients. The “black box effect” refers to recommendations generated by AI algorithms without clear explanations. It means that although the model may produce accurate predictions, it is often unclear how or why a given output is reached. This opacity poses serious concerns in emergency surgery, where clinical decisions must be rapidly justified and clearly communicated. Explainable AI (XAI) seeks to address this by providing interpretable decision-making models, ensuring that clinicians can understand and validate AI-driven recommendations before acting upon them [19]. XAI principles are particularly relevant for Generative Pretrained Transformers (GPTs) and other deep learning models, which, despite their impressive capabilities, often lack interpretability and transparency [20,21,22].

In emergency settings, where time is limited and decisions carry high stakes, explainability becomes even more critical. Surgeons must be able to interpret and, if needed, challenge AI recommendations. Emerging solutions include visual heatmaps, logical flow diagrams, and probabilistic reasoning layers that clarify the rationale behind predictions [19,20,21,22].

Despite these advances, current explainability tools are often not integrated into clinical interfaces.

### 4.2. Bias and Equity in AI Algorithms

AI algorithms are trained on historical datasets which may inherently reflect existing biases in healthcare and data quality. If training datasets have an underrepresentation of certain racial, socioeconomic, or gender groups, AI models risk perpetuating these disparities rather than mitigating them [23]. The concept of “health data poverty” highlights how certain populations remain underrepresented in medical research, potentially leading to biased AI recommendations [24]. Addressing these biases requires large, diverse, and inclusive datasets, rigorous validation, and continuous monitoring of AI performance across different patient populations [25,26].

The General Data Protection Regulation (GDPR), introduced in the European Union (EU) in 2016, primarily addresses data security and patient privacy but does not directly mitigate biases or underrepresentation in AI training datasets [27]. While regulatory measures safeguard patient confidentiality, additional frameworks are needed to promote ethical research practices and ensure AI-driven surgical innovations benefit all patient populations equitably. Ensuring compliance with Health Insurance Portability and Accountability Act (HIPAA) security standards is crucial for the protection of Electronic Health Records (EHRs), requiring AI technologies to align with evolving regulatory frameworks [28].

### 4.3. Data Protection and Privacy

Given the complexity of multidisciplinary healthcare, curating accurate, representative datasets requires comprehensive efforts.

One significant initiative driving data protection and transparency is the **FAIR** guiding principles, which emphasize **F**indability, **A**ccessibility, **I**nteroperability, and **R**eusability. These principles are essential in medical Big Data, promoting not only security but also data reproducibility, validation, and generalizability [29]. Further challenges include vulnerabilities to cybersecurity threats such as malware and hacking, posing risks to AI-driven interfaces. One prospective mode of safety precautions for these technological interfaces is an AI “trustworthy architecture that uses decentralized blockchain characteristics such as smart contracts and trust oracles” [30].

In surgery, challenges related to video data storage further complicate AI integration, particularly regarding compliance with privacy regulations. The integration of AI into surgical perioperative decision-making heavily relies on the availability and quality of surgical video data. However, managing and storing video data presents several challenges that can impact the effectiveness of AI applications, such as the following [31]:

- **Storage Capacity and Infrastructure:**
  1. *High Data Volume:* Surgical procedures, especially those recorded in high-definition formats, generate substantial amounts of data. Continuous recording of all surgeries can quickly exceed existing storage capacities, necessitating significant investments in scalable storage solutions.
  2. *Cost Implications:* Maintaining and upgrading storage infrastructure to accommodate the growing volume of video data can be financially burdensome for healthcare institutions, particularly those with limited resources.
- **Data Management and Accessibility:**
  1. *Efficient Retrieval:* As the volume of stored video data increases, implementing effective data management systems becomes essential to ensure that relevant videos can be easily retrieved for analysis and review.
  2. *Standardization Issues:* Variations in video formats, annotations, and metadata can complicate data integration and analysis, underscoring the need for standardized protocols in video recording and storage.
- **Legal and Ethical Considerations:**
  1. *Patient Privacy:* Surgical videos often contain sensitive patient information. Ensuring compliance with data protection regulations, such as GDPR and HIPAA, is crucial to safeguard patient privacy and maintain trust.
  2. *Consent and Data Ownership:* Clarifying issues related to informed consent for recording and using surgical videos, as well as determining data ownership, is essential to address ethical and legal concerns.

### 4.4. Surgical Data Quality

Enhancing the quality of surgical video data to feed AI algorithms is essential for an effective AI integration in perioperative decision-making. Proper annotation, expert validation, and structured labeling ensure that AI models are trained on clinically relevant data, improving accuracy and reliability. However, challenges persist due to a lack of uniform annotation guidelines and standardization. Differences in terminology and classification methods among surgeons and across institutions create inconsistencies that hinder algorithm development and cross-center generalizability.

To mitigate these issues, the implementation of standardized frameworks—such as the SAGES (Society of American Gastrointestinal and Endoscopic Surgeons) consensus on surgical video structuring—is crucial [31]. Manual annotation is time-consuming and subject to inter-observer variability, even among experienced surgeons. Hybrid models that combine computer vision (CV)-generated pre-annotations with expert review can reduce workload while maintaining high precision [8,9,32].

To improve annotation quality, standardized frameworks should be implemented. Hierarchical annotation models that categorize surgical steps into structured stages (e.g., incision, dissection, hemostasis, closure) facilitate AI learning and interpretation. Segmentation-based labeling helps AI models differentiate critical anatomical landmarks, such as Calot’s Triangle in laparoscopic cholecystectomy [10]. Additionally, adopting common surgical taxonomies as recommended by SAGES and EAES (European Association for Endoscopic Surgery) ensures consistency across multiple institutions [33,34]. Beyond annotation, expert validation and quality control play a crucial role in ensuring an AI system’s reliability. A multi-tiered review process—where junior surgeons provide initial annotations, senior surgeons refine them, and AI-assisted correction is applied—enhances dataset accuracy. Crowdsourced labeling platforms, where multiple experts collaboratively annotate large datasets, can further improve precision and efficiency [33]. A study by Hong et al. emphasizes the importance of expert-generated annotations in surgical phase recognition. The researchers observed that discrepancies in annotations, even among experts, can affect the generalization performance of Convolutional Neural Networks (CNNs). By implementing a rigorous annotation process involving multiple specialists, they achieved improved performance in surgical phase recognition models [35].

Emerging collaborative models, such as multi-tiered expert review and crowdsourced labeling platforms, have demonstrated potential. For example, the Annotated Videos of Open Surgery (AVOS) dataset, created through crowd-annotated surgical videos, enabled the development of AI systems capable of interpreting complex intraoperative behavior in real time [35,36].

Advancements in deep learning, including the use of architectures like You Only Look Once (YOLO) v3 and Mask Region-based Convolutional Neural Network (R-CNN), have enabled automated detection of anatomical structures and surgical instruments, accelerating annotation processes [37,38]. Natural Language Processing (NLP) techniques can further enhance data structuring by linking narrative operative reports to corresponding video events [11,39].

Refining classification systems through contextual metadata tagging—including surgeon expertise, intraoperative complications, and patient comorbidities—can improve model generalizability and clinical relevance. By integrating structured annotation, validated expert review, and AI-assisted tools, healthcare systems can build reproducible, unbiased datasets to support the safe and effective use of AI in emergency surgery.

### 4.5. Real-Time Data and Workflow in the Operating Room

The integration of AI into the surgical environment is reshaping operating room (OR) workflows—particularly in emergency surgery, where efficiency, precision, and adaptability are critical. Effective OR management involves balancing multiple factors such as estimating case duration, coordinating staff, prioritizing patients based on urgency, and allocating resources efficiently [40]. Delays or inefficiencies in these processes can increase complications and worsen outcomes, highlighting the need for intelligent workflow optimization tools [41].

AI-driven systems are addressing these challenges by leveraging ML, real-time data analytics, and CV to enhance intraoperative monitoring, resource utilization, and patient safety. In unpredictable emergency settings, these tools can dynamically adjust schedules and prioritize cases using classification systems like the New Timing in Acute Care Surgery (New TACS), which stratify patients by disease severity and timing needs [42].

A notable innovation is the OR Black Box System—an AI-powered platform inspired by aviation’s black box concept. It records synchronized data streams, including audio, video, vitals, and equipment metrics, to identify risks and promote quality improvement. The system de-identifies visual and audio data to preserve team privacy while enabling constructive feedback on technical and non-technical performance [43,44].

Real-world adoption has demonstrated its effectiveness. Stanford Hospital reported improved safety protocols and workflow efficiency, while Toronto General Hospital observed fewer non-technical errors, especially those linked to miscommunication and stress [45,46]. These insights foster continuous professional development and help identify systemic issues, such as equipment failures or protocol deviations, allowing for targeted interventions [47,48].

In parallel, CV-based AI tools are being used to support surgical phase recognition and anatomical landmark identification, enhancing precision and reducing operative times. For instance, AI models trained to detect the “Critical View of Safety” in laparoscopic cholecystectomy have helped reduce bile duct injuries and standardize procedural safety [49]. Instrument tracking systems further assist OR teams by anticipating surgical needs, minimizing delays, and optimizing intraoperative efficiency [50].

While the benefits of AI-enhanced OR workflows are substantial, caution is warranted. Excessive reliance on automation risks undermining clinical autonomy. AI should function as an augmentation—not a replacement—of surgical judgment, maintaining the surgeon’s central role in intraoperative decision-making.

Equity must also be considered. Many AI tools are trained on data from well-resourced centers, limiting their generalizability to underfunded or rural hospitals. Ensuring dataset diversity is essential to avoid amplifying healthcare disparities and to guarantee the safe implementation of AI across various surgical contexts.

Finally, widespread use of AI-based OR monitoring raises ethical concerns around data privacy and ownership. Even with de-identification protocols, unresolved questions persist regarding the long-term storage and use of this data for research or quality improvement. Transparent governance frameworks will be crucial to protect patient and clinician rights while fostering trust in AI-driven innovation.

### 4.6. Informed Consent in Emergency AI-Assisted Surgery

Unlike elective surgeries, emergency procedures often occur under circumstances where obtaining informed consent is challenging. AI integration adds another layer of complexity, as patients may not fully understand its role in their treatment. Studies have indicated that patients prefer transparency regarding AI involvement in their care and show increased trust when AI systems are disclosed and explained [51,52]. Furthermore, evidence suggests that when patients are adequately educated about AI’s role, their acceptance and confidence in AI-assisted procedures significantly improve, highlighting the necessity for transparency in AI deployment [53]. Beyond informed consent, AI integration in surgery must align with core ethical principles, particularly the respect for patient autonomy. Ethical concerns arise when AI-driven decisions conflict with individual patient values, potentially leading to recommendations that may not fully reflect personal preferences. This underscores the critical need for AI systems that are not only explainable but also adaptable to patient-centered care. Ensuring that AI remains a supportive tool rather than a substitute for human judgment is essential to maintain ethical integrity in emergency surgical settings [54].

Developing standardized protocols to communicate AI usage to patients or their families is essential for maintaining ethical standards in emergency surgery. As AI continues to evolve in emergency surgical practice, healthcare professionals must advocate for ethical frameworks that prioritize patient engagement, informed consent, and shared decision-making.

### 4.7. Regulatory and Legal Frameworks

Regulatory frameworks for AI in healthcare vary widely across jurisdictions, as shown in Table 2.

The European Union’s General Data Protection Regulation (GDPR) sets robust standards for data protection and consent, yet it does not directly address key issues such as algorithmic bias or dataset representativeness [27]. In parallel, the U.S. Food and Drug Administration (FDA) has authorized several AI-based surgical tools and continues to update its regulatory pathways for autonomous systems, focusing on safety and performance [55].

In recognition of the need for global alignment, the World Health Organization (WHO) has proposed six core principles for ethical AI governance in health. These pillars emphasize safety, transparency, inclusiveness, human-centered design, and accountability. The WHO also underscores AI’s potential to improve diagnosis, treatment, self-care, and professional training, provided that implementation adheres to rigorous ethical standards [56].

At the legislative level, the European Commission proposed the first comprehensive AI regulation in 2021. This framework adopts a risk-based approach, requiring that high-risk AI systems in healthcare demonstrate transparency, human oversight, non-discrimination, and traceability [57]. Importantly, it reinforces the principle that automation should support—not replace—human judgment to prevent harm.

However, legal requirements and enforcement mechanisms vary widely by region. As a result, the integration of AI into clinical practice remains uneven, with national policies often lacking cohesion. To ethically advance the field, consistent global standards are needed—particularly in emergency surgery, where real-time, cross-institutional collaboration is common.

- The issue of explainability is increasingly central to regulatory efforts. EU legislation, including the GDPR and amended Directive 2011/83 on Consumer Rights, outlines obligations for explainable AI in automated decision-making (e.g., GDPR Articles 13.2(f) and 14.2(g)) [55]. In practice, explainability entails providing clinicians with access to key information such as the following:
- The main features driving the model’s decision;
- All contributing data points;
- How features interact in the model’s logic;
- And, in some cases, the architecture of the model itself [58].

The FDA first approved surgical robots in 2000, and since then, the number of AI/ML-enabled devices has grown dramatically. In 2020, the use of these tools increased by 39% compared to the previous year, with 2023 volumes projected to exceed 30% of all new digital devices [55,59,60,61]. These systems are now used not only to assist surgical procedures but also to support diagnostic accuracy, with the potential to reduce treatment costs by up to 50% and improve health outcomes by 40%.

Nevertheless, regulatory coverage remains limited. Although many AI tools function as decision-support systems, they still pose significant ethical and safety risks—especially when used in high-stakes, autonomous roles. Regulations vary across manufacturers, and existing standards do not yet fully address the complexities of moral accountability, real-world variability, or the diverse patient populations served by AI.

To ensure ethical implementation, regulators must prioritize comprehensive documentation, risk assessment, data validation, and transparency. Privacy protections and equitable data quality standards must also be enforced. Given the complexity and sensitivity of surgical AI systems—especially in emergency care—a unified, globally harmonized regulatory framework is essential for ensuring both innovation and patient safety.

### 4.8. Liability in AI-Assisted Complications: Who Is Responsible?

The introduction of AI in emergency surgery brings complex medico-legal challenges, regarding who is liable when AI-driven decisions contribute to a complication or negative outcomes. It is not clear who is accountable—the surgeon, the hospital, the AI developer, or the manufacturer—when an AI-assisted system (ML) makes an incorrect recommendation or an AI-automated tool malfunctions [62,63].

AI’s role in emergency decision-making must be contextualized based on its intended role and defined as follows (Table 3):

- **Supportive AI**, aimed to support clinical decision-making;
- **AI-assisted decision-making**, which provides semi-autonomous guidance;
- **Autonomous AI in predefined tasks**, which automates specific surgical steps.

Liability in AI-based tool complications can be categorized into three main scenarios according to the degree of autonomy of the AI device implemented, as summarized in Table 4 [63]:

Current FDA and EU regulations do not define liability for AI-assisted errors in medicine, leading to grey areas in litigation.

In clinical practice, patients might need to explicitly consent to AI-assisted decisions and to be aware the AI is involved in the management of his/her surgical disease.

Furthermore, AI should explain why it made a certain decision providing black box AI systems clearer validation frameworks.

Future policies may introduce hybrid liability models, where hospitals, surgeons, and AI vendors share responsibility based on case specifics.

## 5. Call to Action and Clinical Recommendations for Ethical AI Implementation in Emergency Surgery

Significant concerns remain regarding the opacity of AI algorithms and the risk of automation bias, where surgeons may overly rely on AI recommendations without independent clinical assessment. This raises critical ethical and practical questions: “Should AI be permitted to override human judgment in high-stakes surgical emergencies? How can we ensure that AI serves as an augmentation of clinical expertise rather than a replacement?”

To address these concerns, it is essential to establish clear lines of responsibility and liability when AI systems contribute to emergency medical decisions. Defining accountability ensures both patient safety and legal protection for healthcare providers. Additionally, caution must be exercised to prevent over-reliance on AI, particularly when it challenges human autonomy in critical decision-making.

To optimize the responsible and effective integration of AI in emergency surgery, actions are required according to the following:

- **Enhancing AI Transparency**—Prioritizing the development of explainable AI (XAI) models to improve interpretability, ensuring that healthcare providers can critically assess and validate AI-generated recommendations.
- **Developing Clear Communication Protocols**—Standardizing the disclosure of AI involvement in patient care to maintain trust and uphold patient autonomy.
- **Mitigating Bias in AI Training Data**—Ensuring that AI training datasets are diverse and representative of all patient populations to prevent the exacerbation of health disparities.
- **Aligning AI with Patient-Centered Care**—Designing AI systems that integrate ethical considerations and patient values into their decision-making frameworks.
- **Strengthening Regulatory Oversight**—Establishing comprehensive legal frameworks to define AI accountability, enhance data protection, and uphold ethical standards in emergency surgical applications.

While XAI techniques hold great promise, they are not yet widely integrated into clinical user interfaces. Future AI systems must prioritize interpretability by design to foster trust, support adoption, and meet evolving transparency and accountability standards [64].

At present, no single global framework fully harmonizes AI regulation and data protection. However, foundational efforts are underway. In the European Union, the General Data Protection Regulation (GDPR) establishes strong rules for data privacy and consent. The proposed EU AI Act complements this with a risk-based approach, mandating transparency, human oversight, and reliability for high-risk AI systems.

In the United States, the FDA offers evolving guidance on AI/ML-enabled medical devices, with a focus on safety and effectiveness, though a unified legal structure equivalent to the EU AI Act is still lacking.

Globally, the WHO has outlined six pillars for ethical AI in health, emphasizing human-centered design, accountability, and fairness. Other efforts, such as the OECD AI Principles and the Global Partnership on AI (GPAI), aim to promote regulatory alignment, though practical implementation—particularly around cross-border data use—remains a major hurdle [65,66].

These legal and institutional differences impact consent processes, algorithm transparency, and equitable access to AI technologies. These challenges are particularly pressing in emergency surgery, where timely decision-making often depends on rapid data exchange among professionals across institutions and international borders. In low- and middle-income countries, limited regulatory infrastructure and technological capacity may hinder the ethical deployment of AI, potentially widening global health disparities.

To enable responsible innovation, developing interoperable legal and technical standards that ensure strong data protection without impeding progress is essential.

By addressing these issues, AI can fulfill its potential to enhance surgical decision-making, improve workflows, and deliver safer, more equitable care in emergency settings [12,67,68,69,70,71,72,73].

Table 5 summarizes the core ethical and regulatory concerns identified in this review and proposes actionable recommendations to guide the safe and effective implementation of AI in emergency surgery.

## 6. Conclusions

The adoption of AI in emergency surgery continues to face significant ethical challenges, including issues of transparency, accountability, data quality and security, and bias mitigation. Although AI is increasingly recognized as a transformative tool in healthcare, regulatory frameworks remain fragmented, and comprehensive ethical guidelines are still evolving.

Responsible integration of AI into emergency surgical practice demands multidisciplinary collaboration among surgeons, clinicians, data scientists, ethicists, and policymakers. Key priorities include mitigating bias in training datasets, strengthening informed consent processes, and establishing robust governance frameworks to uphold trust in AI-assisted decision-making. Additionally, ensuring equitable access to technology, providing clinicians with appropriate training, and fostering active stakeholder engagement will be critical for the safe adoption of AI in surgical workflows.

Future research should focus on implementation science to ensure that AI technologies are generalizable, representative, and adaptable across diverse patient populations and healthcare settings. Notably, most of the current literature and datasets originate from high-income countries, creating a significant gap in understanding how AI can be safely and ethically deployed in low- and middle-income contexts, where resource limitations pose unique challenges.

We strongly advocate for more inclusive, globally coordinated research efforts to ensure that AI tools are equitable, scalable, and responsive to varied healthcare environments. Moreover, empirical validation studies—such as clinical trials and real-world implementation research—should be planned to assess the safety, effectiveness, and ethical implications of AI tools in emergency surgical settings.

Ultimately, AI should not replace human expertise but rather serve as a complementary tool that enhances clinical decision-making while preserving professional judgment—especially in high-stakes emergency scenarios. With ethical oversight, responsible governance, and ongoing refinement, AI has the potential to meaningfully transform emergency surgical care.

## Figures and Tables

**Table 1:** Summary of key studies included in the narrative review.

Authors (Year)	Study Focus	Setting	Key Ethical or Clinical Implications
**Panossian et al. (2025) [1]**	Validation of AI-based risk calculator in emergency laparotomy	Emergency Surgery	Accountability, clinical reliability
**Gebran et al. (2022) [2]**	AI tool to predict ICU need after emergency surgery	Emergency Surgery	Predictive validity, clinical utility
**Elhaddad and Hamam** **(2024) [3]**	Review on AI-driven clinical decision-support systems	General Healthcare	Potential vs. limitations of AI in decision-making
**Capelli et al. (2023) [4]**	White paper on ethics and trust in AI-assisted surgery	Clinical Surgery	Trustworthiness, transparency
**Cobianchi et al. (2022) [5]**	Ethical dilemmas of AI in surgery	General Surgery	Bias, autonomy, data governance
**Hashimoto et al. (2018) [7]**	Promises and perils of AI in surgery	General Surgery	Technological optimism, black box concerns
**Mascagni et al. (2022) [8]**	AI for surgical safety: critical view assessment	Laparoscopic Surgery	AI-driven safety enhancement
**Mascagni et al. (2021) [9]**	Computer vision for detecting surgical events	Laparoscopic Surgery	Video annotation and decision support
**Shinozuka et al. (2022) [10]**	AI for surgical phase recognition	Laparoscopic Surgery	Workflow optimization, data use
**Madani et al. (2022) [11]**	Semantic segmentation for intraoperative guidance	Laparoscopic Surgery	Surgical anatomy identification
**De Simone et al. (2022) [12]**	Global survey about AI-assisted implementation in emergency surgical practices	Emergency Surgery	Knowledge, attitudes, perspectives, and barriers perceived by emergency surgeons to AI-driven tool implementation in the emergency setting

**Table 2:** Key global regulatory frameworks related to AI in emergency surgery. FDA: U.S. Food and Drug Administration; GDPR: General Data Protection Regulation; EU: European Union; MHRA: Medicines and Healthcare products Regulatory Agency; NMPA: National Medical Products Administration.

Regulatory Body	Region	Key AI Guidelines	Major Challenges
**FDA (U.S.)**	United States	AI/ML-based surgical tools must undergo **premarket approval (PMA)** or **510(k) clearance**.	Lack of standardized AI-specific rules; most AI tools classified as **decision-support** rather than **autonomous systems**.
**GDPR (EU)**	European Union	Focuses on **data privacy**, **informed consent**, and **algorithmic transparency**.	**No clear AI-specific medical regulations**; AI explainability requirements are still evolving.
**EU AI Act**	European Union	First global AI regulatory framework, categorizing AI applications based on **risk levels** (minimal, high, unacceptable).	**Surgical AI could be classified as “high risk”,** requiring stringent validation and real-time monitoring.
**WHO AI Ethics Framework**	Global	Calls for **ethical AI integration** with principles of **trustworthiness, fairness, and transparency**.	Non-binding recommendations; lacks enforcement mechanisms for individual countries.
**MHRA (UK)**	United Kingdom	Requires **AI as a Medical Device (AIaMD)** to meet CE marking for safety and performance.	**Post-market AI monitoring is weak**, making it difficult to detect AI-related adverse events in real time.
**China NMPA AI Regulations**	China	Encourages AI in healthcare but requires **strict cybersecurity measures and data localization**.	AI models must be **trained on Chinese patient data,** limiting generalizability across global populations.

**Table 3:** Levels of AI integration in emergency surgical decision-making and associated ethical considerations. This table outlines the progressive levels of AI integration in emergency surgery, from assistive tools to experimental full automation, with examples and ethical implications for each stage.

AI Role	Description	Case Example in Emergency Surgery	Ethical and Practical Considerations
**AI as a Decision-Support Tool (Assistive AI)**	Provides real-time data analysis, risk assessment, and decision-making support without making autonomous choices.	POTTER Calculator predicts postoperative mortality and complications in emergency general surgery, improving triage and resource allocation.	Surgeons retain full control; AI acts as an augmentation tool to reduce cognitive load. Trust and interpretability (XAI) are key.
**AI-Assisted Decision-Making (Semi-Autonomous AI)**	AI suggests interventions based on real-time surgical video, patient data, or intraoperative findings. The final decision remains with the surgeon.	Computer vision evaluates intestinal perfusion via ICG fluorescence imaging, aiding in anastomotic leak prevention.	AI requires explainability and surgeon oversight. If AI misinterprets perfusion, who is responsible? There is a lack of clear medico-legal guidelines.
**AI-Enabled Automation (Task-Specific AI)**	AI executes predefined actions autonomously within a controlled scope.	AI-powered automatic staplers (e.g., Medtronic Signia™) adjust stapling depth and compression based on tissue thickness sensors, reducing human error in colorectal anastomosis.	This event requires human intervention in case of AI failure. If a staple misfires, is it a surgeon’s or manufacturer’s responsibility? There are not clear regulations on it.
**Fully Autonomous AI Surgery (Future Concept)**	AI performs entire surgical procedures autonomously without direct human input.	STAR Robot (Smart Tissue Autonomous Robot) successfully performed soft-tissue anastomosis in a porcine model with greater precision than human surgeons.	Not yet ethically or legally acceptable in humans. Requires new regulatory frameworks to define accountability and patient consent.

**Table 4:** Legal liability scenarios in AI-assisted emergency surgical practices. This table presents illustrative liability scenarios based on different degrees of AI involvement in emergency surgery, emphasizing shared responsibility between clinicians, developers, and manufacturers.

Scenario	Example	Liability Considerations
**AI as a Decision-Support Tool (Assistive AI)**	POTTER calculator underestimates a patient’s risk, leading to an anastomosis that fails instead of a safer Hartmann’s procedure.	The surgeon retains final decision-making authority, so primary liability falls on the clinician. However, if AI models were trained on biased datasets, legal responsibility may extend to developers and institutions.
**AI-Assisted Surgical Decision-Making**	AI misinterprets ICG fluorescence imaging and fails to detect ischemia before an anastomosis, leading to a leak.	If the surgeon over-relied on AI despite conflicting clinical findings, they may share liability. However, if AI misdiagnosis results from algorithmic failure, the AI vendor could be held accountable under product liability laws.
**AI-Enabled Automation (Task-Specific AI)**	AI-powered stapler malfunctions and misfires, causing anastomotic dehiscence.	Product liability law applies—manufacturer is responsible for device failure unless the surgeon misused the device against recommendations. AI safety validation is crucial.

**Table 5:** Key clinical recommendations for ethical AI implementation in emergency surgery.

Ethical Issue	Clinical Recommendation
**Informed Consent in Emergency Settings**	Implement standardized protocols to inform patients or their proxies about AI involvement during emergency procedures.
**Explainability and Transparency**	Adopt explainable AI tools to ensure clinicians can interpret and validate system outputs in real time.
**Bias and Equity in AI Models**	Ensure diverse and representative datasets are used to train AI, reducing bias across populations.
**Liability in AI-Assisted Complications**	Develop shared responsibility models between surgeons, institutions, and AI developers for adverse outcomes.
**Surgical Video Data Governance**	Follow ethical and legal frameworks (e.g., GDPR, HIPAA) for storage, use, and consent of surgical video data.
**Workflow Integration in Emergency Surgery**	Use AI tools to support—rather than replace—surgeon decision-making; maintain human oversight during intraoperative use.
**Data Quality and Annotation**	Promote the prospective collection of high-quality clinical data and intraoperative images through standardized acquisition protocols, expert-validated annotation, and interoperability between centers.

## Data Availability

Not applicable.

## Abbreviations

AI	Artificial Intelligence
ML	Machine Learning
DL	Deep Learning
CV	Computer Vision
CNNs	Convolutional Neural Networks
R-CNN	Region-based Convolutional Neural Network
FDA	U.S. Food and Drug Administration
GDPR	General Data Protection Regulation
EU	European Union
MHRA	Medicines and Healthcare products Regulatory Agency
NMPA	National Medical Products Administration

## References

[1] Panossian V.S., Argandykov D., Arnold S.C., Gebran A., Paranjape C.N., Hwabejire J.O., DeWane M.P., Velmahos G.C., Kaafarani H.M., POTTER Validation Group (2025). Validation of Artificial Intelligence-Based POTTER Calculator in Emergency General Surgery Patients Undergoing Laparotomy: Prospective, Bi-Institutional Study. J. Am. Coll. Surg..

[2] Gebran A., Vapsi A., Maurer L.R., El Moheb M., Naar L., Thakur S.S., Sinyard R., Daye D., Velmahos G.C., Bertsimas D. (2022). POTTER-ICU: An artificial intelligence smartphone-accessible tool to predict the need for intensive care after emergency surgery. Surgery.

[3] Elhaddad M., Hamam S. (2024). AI-Driven Clinical Decision Support Systems: An Ongoing Pursuit of Potential. Cureus.

[4] Capelli G., Verdi D., Frigerio I., Rashidian N., Ficorilli A., Grasso V., Majidi D., Gumbs A.A., Spolverato G., Artificial Intelligence Surgery Editorial Board Study Group on Ethics (2023). White paper: Ethics and trustworthiness of artificial intelligence in clinical surgery. Artif. Intell. Surg..

[5] Cobianchi L., Verde J.M., Loftus T.J., Piccolo D., Dal Mas F., Mascagni P., Garcia Vazquez A., Ansaloni L., Marseglia G.R., Massaro M. (2022). Artificial Intelligence and Surgery: Ethical Dilemmas and Open Issues. J. Am. Coll. Surg..

[6] Braun V., Clarke V. (2019). Reflecting on reflexive thematic analysis. Qual. Res. Sport Exerc. Health.

[7] Hashimoto D.A., Rosman G., Rus D., Meireles O.R. (2018). Artificial Intelligence in Surgery: Promises and Perils. Ann. Surg..

[8] Mascagni P., Vardazaryan A., Alapatt D., Urade T., Emre T., Fiorillo C., Pessaux P., Mutter D., Marescaux J., Costamagna G. (2022). Artificial Intelligence for Surgical Safety: Automatic Assessment of the Critical View of Safety in Laparoscopic Cholecystectomy Using Deep Learning. Ann. Surg..

[9] Mascagni P., Alapatt D., Urade T., Vardazaryan A., Mutter D., Marescaux J., Costamagna G., Dallemagne B., Padoy N. (2021). A Computer Vision Platform to Automatically Locate Critical Events in Surgical Videos: Documenting Safety in Laparoscopic Cholecystectomy. Ann. Surg..

[10] Shinozuka K., Turuda S., Fujinaga A., Nakanuma H., Kawamura M., Matsunobu Y., Tanaka Y., Kamiyama T., Ebe K., Endo Y. (2022). Artificial intelligence software available for medical devices: Surgical phase recognition in laparoscopic cholecystectomy. Surg. Endosc..

[11] Madani A., Namazi B., Altieri M.S., Hashimoto D.A., Rivera A.M., Pucher P.H., Navarrete-Welton A., Sankaranarayanan G., Brunt L.M., Okrainec A. (2022). Artificial Intelligence for Intraoperative Guidance: Using Semantic Segmentation to Identify Surgical Anatomy During Laparoscopic Cholecystectomy. Ann. Surg..

[12] De Simone B., Abu-Zidan F.M., Gumbs A.A., Chouillard E., Di Saverio S., Sartelli M., Coccolini F., Ansaloni L., Collins T., Kluger Y. (2022). Knowledge, attitude, and practice of artificial intelligence in emergency and trauma surgery, the ARIES project: An international web-based survey. World J. Emerg. Surg..

[13] Rodgers C.M., Ellingson S.R., Chatterjee P. (2023). Open Data and transparency in artificial intelligence and machine learning: A new era of research. F1000Research.

[14] Angelos P. (2009). Complications, Errors, and Surgical Ethics. World J. Surg..

[15] Fosch-Villaronga E., Khanna P., Drukarch H., Custers B. (2023). The Role of Humans in Surgery Automation. Int. J. Soc. Robot..

[16] Kim Y.S., Park S.H., Lee I.Y., Son G.M., Baek K.R. (2024). AI-driven automatic compression system for colorectal anastomosis. J. Robot. Surg..

[17] Habli I., Lawton T., Porter Z. (2020). Artificial intelligence in health care: Accountability and safety. Bull. World Health Organ..

[18] Adegbesan A., Akingbola A., Aremu O., Adewole O., Amamdikwa J.C., Shagaya U. (2024). From Scalpels to Algorithms: The Risk of Dependence on Artificial Intelligence in Surgery. J. Med. Surg. Public Health.

[19] Shahbazi Z., Byun Y.C. (2022). Analysis of the Security and Reliability of Cryptocurrency Systems Using Knowledge Discovery and Machine Learning Methods. Sensors.

[20] Brożek B., Furman M., Jakubiec M., Kucharzyk B. (2023). The black box problem revisited. Real and imaginary challenges for automated legal decision making. Artif. Intell. Law.

[21] Iserson K., Baker E., Bissmeyer P., Derse A. (2024). Artificial Intelligence in the ED: Ethical Issues. ACEP Now.

[22] Hassija V., Chamola V., Mahapatra A., Singal A., Goel D., Huang K., Scardapane S., Spinelli I., Mahmud M., Hussain A. (2024). Interpreting Black-Box Models: A Review on Explainable Artificial Intelligence. Cogn. Comput..

[23] Agarwal R., Bjarnadottir M., Rhue L., Dugas M., Crowley K., Clark J., Gao G. (2023). Addressing algorithmic bias and the perpetuation of health inequities: An AI bias aware framework. Health Policy Technol..

[24] Prien C., Lincango E.P., Holubar S.D. (2023). Big Data in Surgery. Surg. Clin. N. Am..

[25] Cross J.L., Choma M.A., Onofrey J.A. (2024). Bias in medical AI: Implications for clinical decision-making. PLoS Digit. Health.

[26] Ueda D., Kakinuma T., Fujita S., Kamagata K., Fushimi Y., Ito R., Matsui Y., Nozaki T., Nakaura T., Fujima N. (2024). Fairness of artificial intelligence in healthcare: Review and recommendations. jpn J. Radiol..

[27] The Impact of the General Data Protection Regulation (GDPR) on Artificial Intelligence. https://www.europarl.europa.eu/RegData/etudes/STUD/2020/641530/EPRS_STU(2020)641530_EN.pdf.

[28] World Health Organization (2023). WHO Calls for Safe and Ethical AI for Health. https://www.who.int/news/item/16-05-2023-who-calls-for-safe-and-ethical-ai-for-health.

[29] Wilkinson M.D., Dumontier M., Aalbersberg I.J., Appleton G., Axton M., Baak A., Blomberg N., Boiten J.W., da Silva Santos L.B., Bourne P.E. (2016). The FAIR Guiding Principles for scientific data management and stewardship. Sci. Data.

[30] van Grinsven M.J.J.P., van Ginneken B., Hoyng C.B., Theelen T., Sanchez C.I. (2016). Fast Convolutional Neural Network Training Using Selective Data Sampling: Application to Hemorrhage Detection in Color Fundus Images. IEEE Trans. Med. Imaging.

[31] Eckhoff J.A., Rosman G., Altieri M.S., Speidel S., Stoyanov D., Anvari M., Meier-Hein L., März K., Jannin P., Pugh C. (2023). SAGES consensus recommendations on surgical video data use, structure, and exploration (for research in artificial intelligence, clinical quality improvement, and surgical education). Surg. Endosc..

[32] Nyangoh Timoh K., Huaulme A., Cleary K., Zaheer M.A., Lavoué V., Donoho D., Jannin P. (2023). A systematic review of annotation for surgical process model analysis in minimally invasive surgery based on video. Surg. Endosc..

[33] Meireles O.R., Rosman G., Altieri M.S., Carin L., Hager G., Madani A., Padoy N., Pugh C.M., Sylla P., Ward T.M. (2021). SAGES consensus recommendations on an annotation framework for surgical video. Surg. Endosc..

[34] Neugebauer E.A., Becker M., Buess G.F., Cuschieri A., Dauben H.P., Fingerhut A., Fuchs K.H., Habermalz B., Lantsberg L., Morino M. (2010). EAES recommendations on methodology of innovation management in endoscopic surgery. Surg. Endosc..

[35] Hong S., Lee J., Park B., Alwusaibie A.A., Alfadhel A.H., Park S., Hyung W.J., Choi M.-K. (2021). Rethinking Generalization Performance of Surgical Phase Recognition with Expert-Generated Annotations. arXiv.

[36] Goodman E.D., Patel K.K., Zhang Y., Locke W., Kennedy C.J., Mehrotra R., Ren S., Guan M., Zohar O., Downing M. (2024). Analyzing Surgical Technique in Diverse Open Surgical Videos With Multitask Machine Learning. JAMA Surg..

[37] Lee J.-D., Chien J.-C., Hsu Y.-T., Wu C.-T. (2021). Automatic Surgical Instrument Recognition—A Case of Comparison Study between the Faster R-CNN, Mask R-CNN, and Single-Shot Multi-Box Detectors. Appl. Sci..

[38] Jiang K., Pan S., Yang L., Yu J., Lin Y., Wang H. (2023). Surgical Instrument Recognition Based on Improved YOLOv5. Appl. Sci..

[39] Sagheb E., Ramazanian T., Tafti A.P., Fu S., Kremers W.K., Berry D.J., Lewallen D.G., Sohn S., Maradit Kremers H. (2021). Use of Natural Language Processing Algorithms to Identify Common Data Elements in Operative Notes for Knee Arthroplasty. J. Arthroplast..

[40] Birkhoff D.C., van Dalen A.S.H.M., Schijven M.P. (2021). A Review on the Current Applications of Artificial Intelligence in the Operating Room. Surg. Innov..

[41] De Simone B., Agnoletti V., Abu-Zidan F.M., Biffl W.L., Moore E.E., Chouillard E., Coccolini F., Sartelli M., Podda M., Di Saverio S. (2024). The Operating Room management for emergency Surgical Activity (ORSA) study: A WSES international survey. Updates Surg..

[42] De Simone B., Kluger Y., Moore E.E., Sartelli M., Abu-Zidan F.M., Coccolini F., Ansaloni L., Tebala G.D., Di Saverio S., Di Carlo I. (2023). The new timing in acute care surgery (new TACS) classification: A WSES Delphi consensus study. World J. Emerg. Surg..

[43] (2023). Tony Peregrin. Black Box Technology Shines Light on Improving OR Safety, Efficiency. https://www.facs.org/for-medical-professionals/news-publications/news-and-articles/bulletin/2023/july-2023-volume-108-issue-7/black-box-technology-shines-light-on-improving-or-safety-efficiency/.

[44] Mascagni P., Padoy N. (2021). OR black box and surgical control tower: Recording and streaming data and analytics to improve surgical care. J. Visc. Surg..

[45] Bai N. (2022). ‘Black Boxes’ in Stanford Hospital Operating Rooms aid Training and Safety. Stanford Medicine News Center.

[46] Jung J.J., Jüni P., Lebovic G., Grantcharov T. (2020). First-year Analysis of the Operating Room Black Box Study. Ann. Surg..

[47] Campbell K.K., Abreu A.A., Zeh H.J., Daniel W.C., Palter V.N., Bishop S.J., Sims S., Odeh J.M., Evans K., Dandekar P. (2024). Using OR Black Box Technology to Determine Quality Improvement Outcomes for In-situ Timeout and Debrief Simulation. Ann. Surg..

[48] Rai A., Beland L., Aro T., Jarrett M., Kavoussi L. (2021). Patient Safety in the Operating Room During Urologic Surgery: The OR Black Box Experience. World J. Surg..

[49] Golany T., Aides A., Freedman D., Rabani N., Liu Y., Rivlin E., Corrado G.S., Matias Y., Khoury W., Kashtan H. (2022). Artificial intelligence for phase recognition in complex laparoscopic cholecystectomy. Surg. Endosc..

[50] Deol E.S., Henning G., Basourakos S., Vasdev R.M.S., Sharma V., Kavoussi N.L., Karnes R.J., Leibovich B.C., Boorjian S.A., Khanna A. (2024). Artificial intelligence model for automated surgical instrument detection and counting: An experimental proof-of-concept study. Patient Saf. Surg..

[51] Park H.J. (2024). Patient perspectives on informed consent for medical AI: A web-based experiment. Digit Health.

[52] Kituuka O., Munabi I.G., Mwaka E.S., Galukande M., Harris M., Sewankambo N. (2023). Informed consent process for emergency surgery: A scoping review of stakeholders’ perspectives, challenges, ethical concepts, and policies. SAGE Open Med..

[53] Teasdale A., Mills L., Costello R. (2024). Artificial Intelligence-Powered Surgical Consent: Patient Insights. Cureus.

[54] Harishbhai Tilala M., Kumar Chenchala P., Choppadandi A., Kaur J., Naguri S., Saoji R., Devaguptapu B. (2024). Ethical Considerations in the Use of Artificial Intelligence and Machine Learning in Health Care: A Comprehensive Review. Cureus.

[55] Artificial Intelligence and Machine Learning (AI/ML)-Enabled Medical Devices. https://www.fda.gov/medical-devices/software-medical-device-samd/artificial-intelligence-and-machine-learning-aiml-enabled-medical-devices.

[56] World Health Organization (2023). WHO Outlines Considerations for Regulation of Artificial Intelligence for Health. https://www.who.int/news/item/19-10-2023-who-outlines-considerations-for-regulation-of-artificial-intelligence-for-health.

[57] (2023). EU AI Act: First Regulation on Artificial Intelligence. Topics European Parliament. https://www.europarl.europa.eu/topics/en/article/20230601STO93804/eu-ai-act-first-regulation-on-artificial-intelligence.

[58] Directive 2011/83/EU of the European Parliament and of the Council of 25 October 2011 on Consumer Rights, Amending Council Directive 93/13/EEC and Directive 1999/44/EC of the European Parliament and of the Council and Repealing Council Directive 85/577/EEC and Directive 97/7/EC of the European Parliament and of the Council Text with EEA Relevance. https://eur-lex.europa.eu/eli/dir/2011/83/oj/eng.

[59] Bibal A., Lognoul M., De Streel A., Frénay B. (2021). Legal requirements on explainability in machine learning. Artif. Intell. Law.

[60] Ahuja A.S. (2019). The impact of artificial intelligence in medicine on the future role of the physician. PeerJ.

[61] Maleki Varnosfaderani S., Forouzanfar M. (2024). The Role of AI in Hospitals and Clinics: Transforming Healthcare in the 21st Century. Bioengineering.

[62] Cestonaro C., Delicati A., Marcante B., Caenazzo L., Tozzo P. (2023). Defining medical liability when artificial intelligence is applied on diagnostic algorithms: A systematic review. Front. Med..

[63] Eldakak A., Alremeithi A., Dahiyat E., El-Gheriani M., Mohamed H., Abdulrahim Abdulla M.I. (2024). Civil liability for the actions of autonomous AI in healthcare: An invitation to further contemplation. Humanit. Soc. Sci. Commun..

[64] Brandenburg J.M., Müller-Stich B.P., Wagner M., van der Schaar M. (2025). Can surgeons trust AI? Perspectives on machine learning in surgery and the importance of eXplainable Artificial Intelligence (XAI). Langenbeck’s Arch. Surg..

[65] OECD AI Principles Overview. https://oecd.ai/en/ai-principles.

[66] The GPAI Initiative and OECD Work on AI Have Joined Forces Under the GPAI Brand to Create an Integrated Partnership. GPAI Work Is Now Available on the OECD AI Policy Observatory. https://gpai.ai/.

[67] De Simone B., Chouillard E., Gumbs A.A., Loftus T.J., Kaafarani H., Catena F. (2022). Artificial intelligence in surgery: The emergency surgeon’s perspective (the ARIES project). Discov. Health Syst..

[68] De Simone B., Di Saverio S. (2022). Invited Commentary: Artificial Intelligence in Surgical Care: We Must Overcome Ethical Boundaries. J. Am. Coll. Surg..

[69] De Simone B., Kluger Y., Moore E.E., Di Saverio S., Sartelli M., Ansaloni L., Coccolini F., Biffl W.L., Catena F. (2021). The WSES: What do we see in the future?. World J. Emerg. Surg..

[70] De Simone B., Abu-Zidan F.M., Saeidi S., Deeken G., Biffl W.L., Moore E.E., Sartelli M., Coccolini F., Ansaloni L., Di Saverio S. (2024). Knowledge, attitudes and practices of using Indocyanine Green (ICG) fluorescence in emergency surgery: An international web-based survey in the ARtificial Intelligence in Emergency and trauma Surgery (ARIES)-WSES project. Updates Surg..

[71] De Simone B., Abu-Zidan F.M., Boni L., Castillo A.M.G., Cassinotti E., Corradi F., Di Maggio F., Ashraf H., Baiocchi G.L., Tarasconi A. (2025). Indocyanine green fluorescence-guided surgery in the emergency setting: The WSES international consensus position paper. World J. Emerg. Surg..

[72] Saeidi H., Opfermann J.D., Kam M., Wei S., Léonard S., Hsieh M.H. (2022). Autonomous robotic laparoscopic surgery for intestinal anastomosis. Sci. Robot..

[73] Shademan A., Decker R.S., Opfermann J.D., Leonard S., Krieger A., Kim P.C. (2016). Supervised autonomous robotic soft tissue surgery. Sci. Transl. Med..

